# European food safety research: An explorative study with funding experts’ consultation

**DOI:** 10.1016/j.heliyon.2023.e22979

**Published:** 2023-11-28

**Authors:** Tamara Stelzl, Nastasia Belc, Nunzia Cito, Veronica M.T. Lattanzio, Celine Meerpoel, Sarah De Saeger, Hanna-Leena Alakomi, Monika Tomaniova, Jana Hajslova, Sophie Scheibenzuber, Michael Rychlik

**Affiliations:** a Technical University of Munich, TUM School of Life Sciences, Department of Life Science Engineering, Chair of Analytical Food Chemistry, Maxiumus-von-Imhof-Forum 2, 85354, Freising, Germany; b National R&D Institute for Food Bioresources, IBA Bucharest, Banesa Ancuta 5, 021102, Bucharest, Romania; c National Research Council of Italy (CNR), Institute of Sciences of Food Production (ISPA), Via Amendola, 122/O, 70126, Bari, Italy; d Ghent University, Department of Bioanalysis, Centre of Excellence in Mycotoxicology and Public Health, Ottergemsesteenweg 460, 9000, Ghent, Belgium; e VTT Technical Research Centre of Finland Ltd., P.O. Box 1000, FIN-02044, VTT, Finland; f University of Chemistry and Technology Prague, Faculty of Food and Biochemical Technology, Department of Food Analysis and Nutrition, Technicka 3, Prague 6, 166 28, Czech Republic

**Keywords:** Food safety, FoodSafety4EU, Research funding, Social lab, Funding experts

## Abstract

The European research area exhibits considerable opacity and fragmentation in food safety research funding and organizational structures, impeding the exploitation of existing research potential across European countries. Given that food safety is inherently linked to the societal challenges of our time, identifying and removing existing barriers to research funding in this area is crucial. Towards investigating this matter, interviews were conducted with funding bodies from six European countries to assess key issues related to research funding in general and food safety in particular. Funding experts were then invited to a workshop to jointly discuss the challenges identified and explore strategies to address them. Evaluation of the food safety research funding situation in selected European countries revealed both convergences and significant differences among national funding bodies. Engaging with funding experts provided invaluable insights into the issues encountered with research funding, such as inadequate call management staff or insufficient research funds, culminating in a set of recommendations for action to remedy the situation.

## Highlights

• European funding for food safety research lacks alignment and harmonization.
• Adequate funding for food safety research secures global food safety and health.
• Strengthening food safety in research funding promotes scientific innovation.
• Consultation with experts' disclosed major shortcomings in research funding systems.
• Need for closer integration and networking of research funding bodies.

## 1 Introduction

Food safety is a cornerstone of our food system, and ensuring food safety is critical at all stages of the food value chain, all the way from farm to fork [1]. In this context, the FoodSafety4EU (FS4EU) project (https://foodsafety4.eu) conducted an explanatory path to support and highlight that food safety is a key issue in the framework of research and innovation (R&I), by providing insights and input for future funding schemes that can include selected priorities and themes. The FS4EU project is a Horizon 2020 project focused to design, develop and release a multi-stakeholder platform for the future European Food Safety System (FSS). It underpins the European Food Safety Authority (EFSA) mission for risk assessment and communication, with the ambition to be a basis for a knowledge center for food safety in Europe. Therefore, a new Food Safety Platform (www.foodsafety4.eu) has been launched in June 2023. The European network currently consists of 23 consortium partners from 12 countries and 50+ supporting partners (Food Safety Authorities, consumer’ associations, research centers, etc.).

As highlighted by ERA-LEARN, a cooperation network and support platform for public-to-public research & innovation partnerships within the European Research Area (ERA) established and supported by the European Commission, a better *“alignment of existing or planned national (and regional) research and innovation strategies, programmes, activities and* funding*”* [2] is mandatory to tackle the major societal challenges of the 21st century by means of initiating and maintaining more collaborative activities along with the formation of strategic and financial alliances. In this context, the term *“alignment”* allows differing interpretations, which was defined by the European Research Area and Innovation Committee's High Level Group for Joint Programming as *“Strategic approach taken by [EU] Member States to modify their national programmes, priorities or activities as a consequence of the adoption of joint research priorities in the context of Joint Programming, with a view to implement changes to improve the efficiency of investment in research at the level of Member States and the ERA”* [2]. With respect to the funding landscape in food systems, that also entails food safety, a study carried out on behalf of the European Commission revealed that the R&I funding ecosystem is highly fragmented, and research funds are widely distributed among several entities and organizational actors, at both the European Union (EU) level and national levels within different EU Member States (MS) [3]. A preceding mapping of public R&I funding opportunities at national and regional level related to food systems conducted by the Standing Committee on Agricultural Research (SCAR) Strategic Working Group on Food Systems in 2018 similarly revealed a strong fragmentation in investments, mostly directed to the primary production of foods and food processing [4].

The activities of the FS4EU project are aimed, among others, at identifying funding opportunities for food safety research and to improve access to funding and reduce prevailing barriers or shortcomings in EU MS and Associated Countries (AC). Despite wide-ranging literature reviews of scientific and policy-related publications conducted within FS4EU, little information could be gathered on funders' grant-making processes, employed in-house practices, theme identification practices or imposed challenges, from both internal and external perspectives, with a particular focus on food safety. By performing structured interviews with different funding representatives and a workshop for information and knowledge exchange, this study aimed to elicit prevailing issues and challenges related to research funding in general with a particular focus on food safety.

## 2 Methods

In this study, two different qualitative methods were used to get further insights into the current state of food safety funding. First, structured interviews using a standardized questionnaire were performed. Second, a 3-h virtual workshop was organized on July 27, 2022, with the survey participants to share experience and knowledge. Both approaches are described in more detail in the following.

### 2.1 Interviews with funding experts

An international expert working group performed structured interviews by use of a standardized questionnaire with 15 questions during April–June 2022 with key funding bodies in six European countries with the aim to create a “*Food Safety Knowledge Network for the alignment of transnational* funding *cycles and research priorities as fundamental part for safe and sustainable food systems*”. The investigations were undertaken as part of a pilot action implemented within the FS4EU project, more specifically within the Social Lab activities of one of four Food Safety Operational Laboratories (FSOLabs) activated within FS4EU [5]. With 23 participants hailing from 12 nations, the FSOLab2 brought together individuals from diverse business sectors, including research, industry/companies, academia, Food Safety Authorities, consumer advocates, action networks, technology & communication services, all mobilized from the FS4EU large European supporting partner network of voluntary membership. A Social Lab is an experimental space for addressing complex, breakthrough societal challenges at a systemic level by bringing together a multidisciplinary team of diverse actors and implementing a pilot intervention in a practical setting as a collaborative effort. Within a set of four installed FS4EU Hubs [5], representing different European regions, distinct national funding bodies were approached. In this context, the Northern Hub mainly embraced countries located in Northern Europe and Baltic states. The Western Hub mostly covered Western European countries. All Mediterranean countries were allocated to the Southern Hub including Adriatic coastal states, whereas the Eastern Hub was comprised of most Central European states. For a simplified visualization of a country's affiliation to an individual Hub, a representation in the four colors red (South Hub), green (North Hub), blue (West Hub) and yellow (East Hub) with corresponding shading was used for the graphical presentation of the interview results gathered from various funding agencies in the framework of the present study. The participation in the interviews was on a voluntary basis and required prior consent and agreement to the data processing within the FS4EU project. Survey respondents, all of whom were over the age of 18, belonged to public/governmental funding agencies or higher education establishments involved in the funding mechanisms at a national level in Belgium (Federal Public Service Health, Food Chain Safety and Environment (*n* = 1); Flanders Innovation and Entrepreneurship (*n* = 1); Department Economy, Science and Innovation of the Flemish government (*n* = 1)); Czech Republic (The Ministry of Agriculture of the Czech Republic (*n* = 1)); Romania (Executive Agency for Higher Education, Research, Development and Innovation (*n* = 1)); Finland (The Ministry of Agriculture and Forestry (*n* = 1)); Italy (University of Parma (*n* = 1)); and Portugal (Foundation for Science and Technology (*n* = 1)). Survey respondents comprised diverse actors from research funding organizations, including Scientific Officers from the research funding management and allocation sector, evaluators of Research & Development projects in food and agriculture, Departmental Officers and representatives from ministries, Senior Officers in the field of international relations and financing of innovation projects. The primary objectives of the interviews were to gain better knowledge on the funding and topics selection processes related to food safety by exploring funding frameworks, mechanisms and decision-making processes.

### 2.2 Workshop for information and knowledge exchange

Following analysis of the survey results, all respondents and/or qualified representatives of the various funding bodies participating in the survey also joined a special workshop held two months later, to discuss the outcomes of the consultations and share experiences on how to better align food safety research funding in the ERA. Further tangible goals of the workshop were:

⁃ to bring together representatives of different (public) funding bodies from several countries;
⁃ to promote dialogue between different funding bodies across state borders;
⁃ to exchange experiences, improve knowledge sharing and transparency in funding matters and practices applied within different funding organizations;
⁃ to identify complementary funding actions to foster collaboration and strategic alliances, and the formation of transnational networking activities;
⁃ to explore and identify examples of best-practices in funding mechanisms to serve as an impetus for discussion and transformation of standing funding processes;
⁃ to discuss and explore possible strategies for overcoming existing bottlenecks in food safety research funding through mutual exchange and as a rationale for formulating recommendations.

The FS4EU “Funding for Food Safety – Workshop to Share Experiences” was attended by seven representatives from funding entities and two external representatives from research and policy. In addition, ten FS4EU project partners engaged in the FSOLab2 activities joined the event. Participation in the workshop was voluntary and necessitated prior written consent, including authorization for further use of the information and data collected during the workshop for project externals. Compliance with research ethics principles and standards was monitored in FSOLab2 by an ethics management team established under the FS4EU project, supported by an external ethics advisor appointed by the project coordination.

The workshop with funding experts comprised three sessions, two of which were mainly dedicated to providing general information about the workshop objectives, the background of the FS4EU project and FSOLab2, and a brief introduction to the *FOODPathS* project [6] and the upcoming European Partnership on Sustainable Food Systems (SFS) for People, Planet & Climate [7] by an invited speaker. The third session of the workshop was devoted to an open round-table discussion guided by a joint reflection on the interview results collected.

## 3 Results of the survey

The standardized questionnaire for the funding experts consisted of 15 questions, which were grouped into six thematic blocks of questions, the results of which are presented hereafter. The original questionnaire is provided in the Supplementary Material.

### 3.1 Funding responsibility

**Fig. 1:**
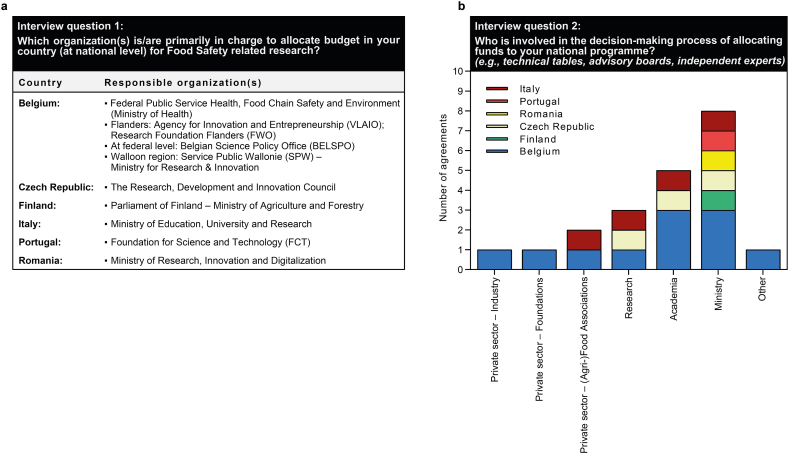
Description of entities involved in research funding allocation at country level (a) and sectors involved in the decision-making process within the funding entity (b).

The first block was dedicated to the description of the organizations in charge for allocating funds and being involved in the decision-making process for assigning funds at their national level (Fig. 1). According to the answers of the respondents, mainly ministries are responsible for allocating the funds (Fig. 1a) and are largely involved in the decision-making processes (Fig. 1b). In addition, three out of the six represented countries stated that research and academia are involved in the decision-making process as well, while (Agri-)Food Associations from the private sector were only named by Belgium and Italy. To a lesser extent, industry and private foundations may also take part in this process.

### 3.2 Funding for food safety

**Fig. 2:**
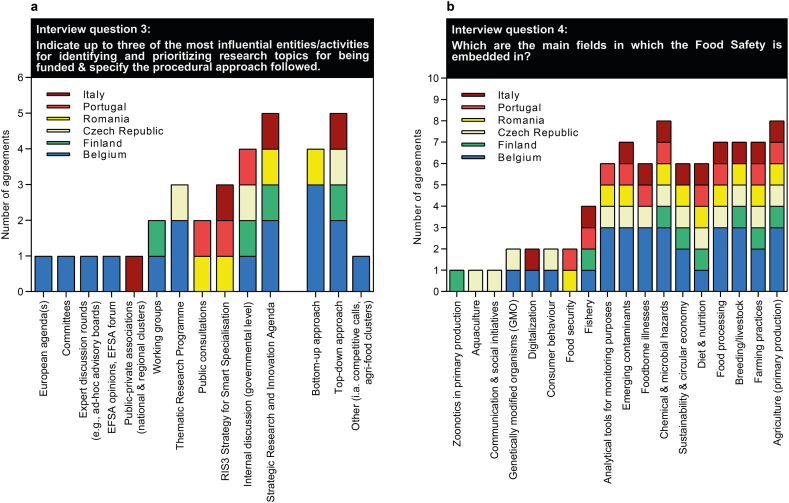
Interview feedback on the most influential entities/activities for identifying and prioritizing research topics (a) and the main fields embedded in the food safety topic (b).

To assess how food safety as a topic is considered in research funding in each country, question block 2 was devoted to sources for prioritization and the integration of food safety into general thematic fields of research funding (Fig. 2). As a result, it became apparent that responding funding experts mainly adopt a top-down approach considering Strategic Research and Innovation Agendas (SRIAs), National Research and Innovation Strategies for Smart Specialization (RIS3), and internal discussions for topics prioritization (Fig. 2a). However, it has to be noted that in most cases, the individual EU MS do not have a specific SRIA for food safety. Public consultations, as well as Thematic Research Programmes and working groups also play a role in several countries, while EFSA opinions, public-private associations, expert discussion rounds, committees and European agendas play a minor role.

The theme food safety is commonly embedded in overarching topics related to primary production (agriculture) or processing (Fig. 2b). In contrast, fields like aquaculture, communication & social initiatives, digitalization, consumer behaviour and food security were identified as less frequent topics for food safety.

However, the MS are advised to follow the objectives of the European Commission. This means that there are already some set priorities in place, which will also be aligned within the SRIA that is currently developed by SCAR [8] as detailed in section 5.2. The scattered nesting of food safety among many themes bears also the disadvantage that there may be some overlap between individual research funding calls, which is discussed further below. But still, some calls address specific hazards such as chemical & microbial hazards, emerging contaminants, and others.

### 3.3 Types of funding on food safety and crisis response

**Fig. 3:**
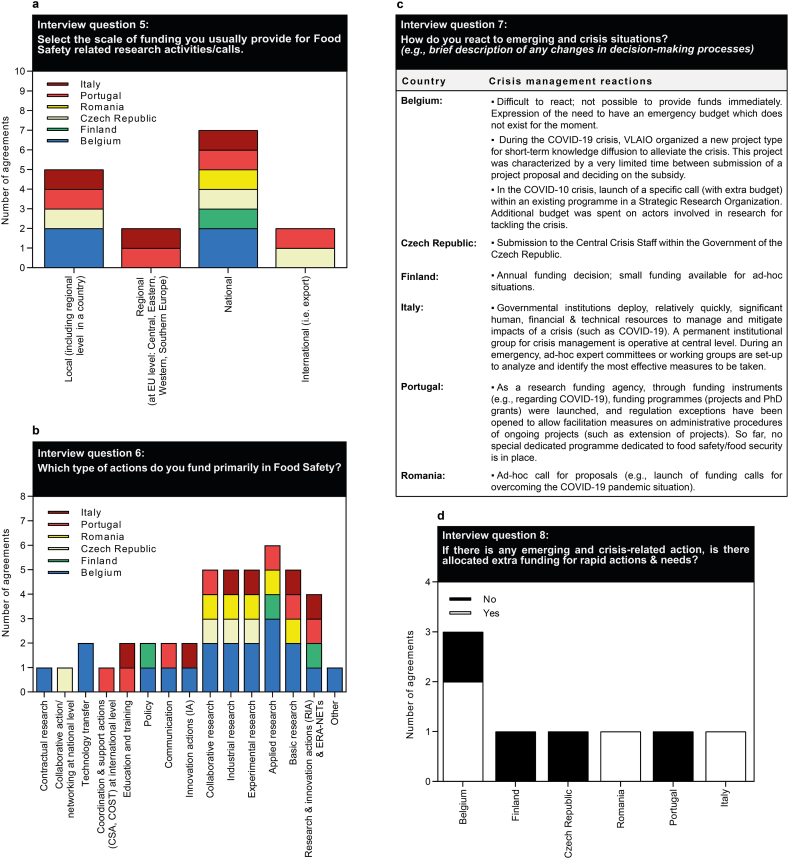
Survey results on the funding scale for food safety research (a), the funded actions (b) as well as the crisis response management in terms of reaction (c) and available extra funding (d).

Question block 3 addressed the types of research funded on food safety and crisis response (Fig. 3). On the one hand, types of actions (basic/applied research, etc.) are highly diverse and mainly funded on a national scale (Fig. 3a/b), while regional and international funding only occurs rarely. Regarding the types of actions, most funding is provided for different research activities, while less funding in the food safety area is allocated to policy and communication/teaching-based actions or technology transfer.

On the other hand, the approach of the funding experts to crises is also highly diverse (Fig. 3c/d). Some of them maintain standing or ad-hoc emergency funding committees, in more than just the food safety arena. Further, the situation in countries is highly scattered between national and regional authorities, and a general European approach is desirable.

### 3.4 Current funding volume

**Fig. 4:**
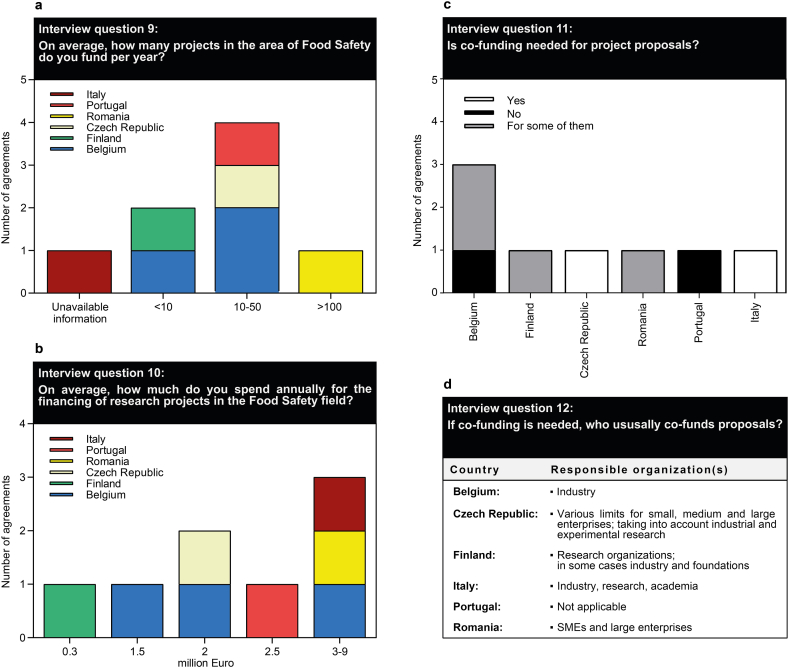
Specifications on the average number of projects funded annually (a) as well as the respective volume of funding (b), funding requirements (c) and co-funding entities (d).

The next question block (Fig. 4) was related to the average number of projects funded annually, the volume of funding, and requirements for getting funding. The replies revealed that the number of funded projects varies widely, with mainly 10–50 food safety focused projects, but up to 100 funded projects in Romania (Fig. 4a) and a total funding volume of maximum €3–9 million per funder (Fig. 4b). However, some participants stated that their funding budget in the food safety field is on average €0.3 million (Finland), €1.5–2 million (Belgium and Czech Republic) and €2.5 million (Portugal), which again highlights the huge fragmentation and disequilibrium within the EU. Additionally, co-funding by industry or small and medium-sized enterprises (SMEs) is required for some countries and some project proposals (Fig. 4c/d). Here, it has to be emphasized that this result has only limited representativeness for the EU.

### 3.5 National and international collaboration

**Fig. 5:**
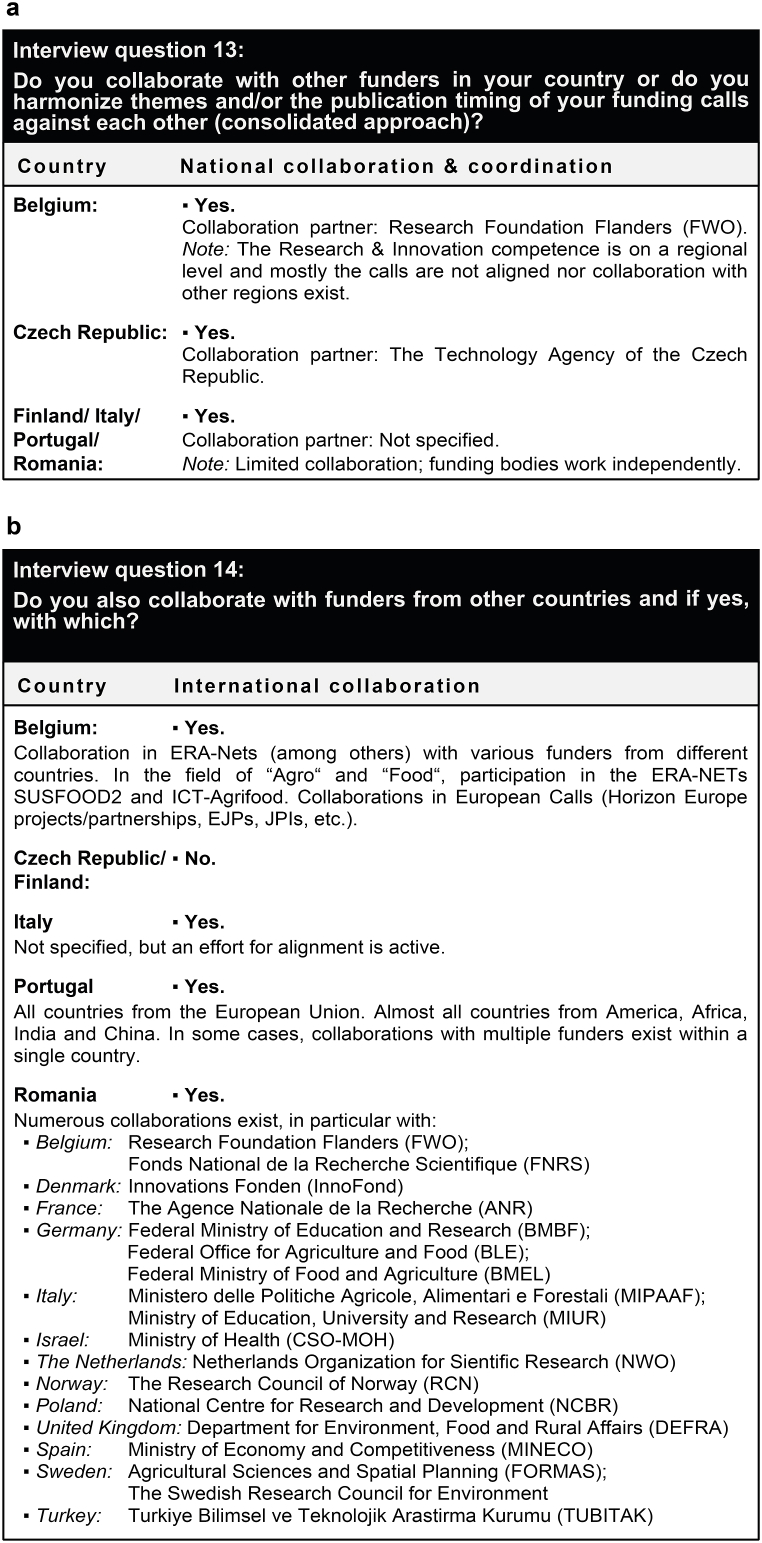
Interview feedback on collaboration activities with other funding entities in the national country (a) and with funding entities from other countries (b).

The fifth question block addressed the collaboration activities with other funders (Fig. 5). It became obvious that collaboration is frequent on a national scale (Fig. 5a), but less prevalent at the international level (Fig. 5b). Opportunities to collaborate on an international level included the European Research Area Networks (ERA-NETs) in the EU's Horizon 2020 funding framework, which are in the process of being transferred into EU Partnerships in the current Horizon Europe framework. Further details on this system are given below.

### 3.6 Funding challenges

**Fig. 6:**
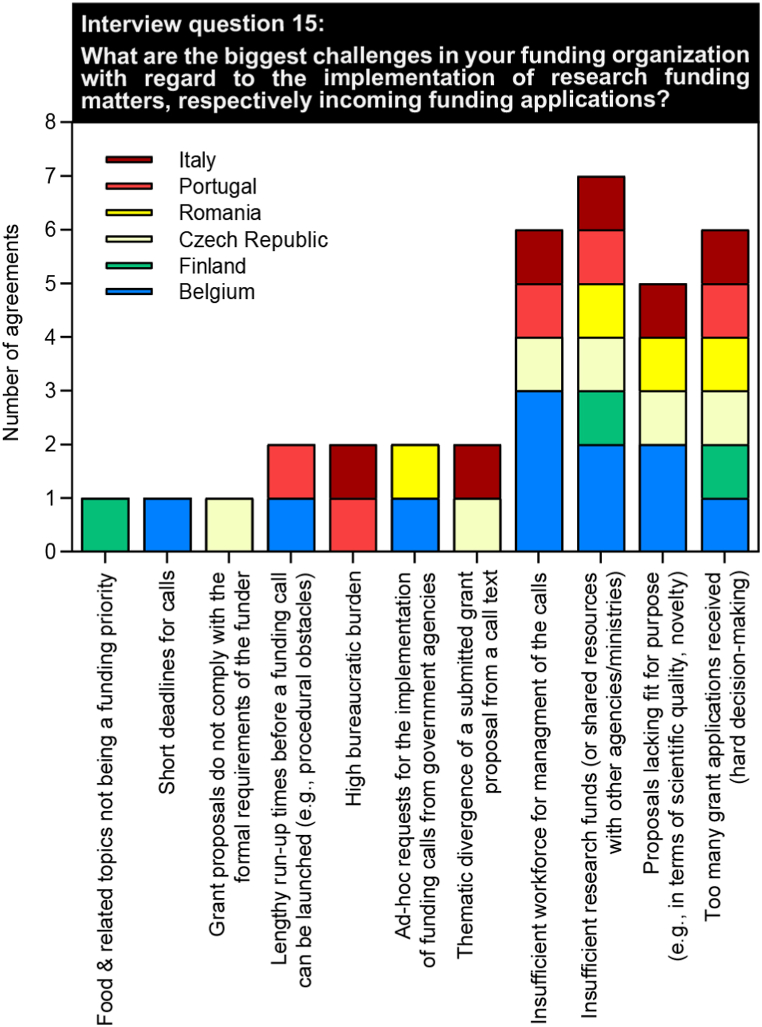
Challenges encountered in implementing and distributing research funds.

The last subject matter was devoted to the challenges in the implementation and distribution of funds from the perspective of the funding experts (Fig. 6). Almost all funding experts stated insufficient workforce for call management and insufficient research funds as the biggest challenges. Regarding the distribution of resources, the funding experts objected to the excessive number of grant applications and the lack of fit for purposes of grant applications received. Other stated challenges were the high bureaucratic burden, procedural obstacles when launching a new call, thematic divergence of a submitted grant proposal from a call, and ad-hoc requests for the implementation of funding calls from government agencies.

## 4 Workshop findings on barriers and recommendations for improving food safety research funding

As a key component of the workshop, a moderated expert discussion was initiated on the basis of the interview results on which major problems and challenges with regard to scientific research funding in general, and food safety in particular, are seen in the respective funding entities and EU countries, respectively where suitable solutions could lie. The following statements provide an outline of the issues raised in the discussions and suggested remedies, which have been rephrased in a more generalized format and supplemented with further information or specific aspects where necessary for better comprehensibility. The main shortcomings related to European research funding and EU research policy, as well as possible suggestions for improvement, were identified as follows:

I. Enhancing traceability in the distribution of financial resources & resolving fragmentation

As evidenced from the survey, in most EU MS/AC, highest subsidies are awarded to researchers by public funding bodies, among others ministries or state authorities (Fig. 1). There are, however, major differences between EU MS/AC, for example, in the partitioning of financial resources across scientific research disciplines, the volume of financial allocations, and the bases and budgeting processes on which research fields and activities are selected to be funded (e.g., top-down or bottom-up approaches, or a mixture of both). This is also a result from question block 2 of our survey. Commonly, research and innovation funding programmes are grounded on national science policies and research agendas, and/or geared towards EU strategies and regulations (e.g., Transparency Regulation (EU) 2019/1381 as a framework for risk assessment and food safety; Farm to Fork Strategy as part of the EU Green Deal) [9,10], and not least may be impacted by external stakeholders and advisers. Public knowledge and understanding of governmental planning processes of national funding programmes and the establishment of national funding priorities often tend to remain opaque, even though governments strive to improve their transparency in political planning processes and the population's and researcher's access to information. In addition, a large number of funding agencies exist in the individual EU MS alongside governmental funding entities and generally operate at a stand-alone basis, making it difficult for the scientific community to gain a deep insight into internal working processes due to their limited transparency. This situation makes it almost impossible to crosscheck main scientific targets and funding objectives across different funding entities, to search for commonalities, and to improve the efficiency of the funding system by better alignment of funding calls at an inter-institutional, but also at the *trans*-national level. In the first instance, there is a demand for closer integration and networking of funding bodies at the regional and national level, in order to jointly coordinate and harmonize activities more effectively. In a second step, *trans*-border networking activities should be established and/or reinforced, and the formation of strategic working alliances as an amalgamation of different funding bodies sponsoring specific research areas, such as for food safety, should be envisaged.

II. Establishment of food safety as a funding discipline in its own right

The search for research funds related to food safety often requires a great deal of time and effort on the part of researchers, especially when it is necessary to study each description of available funding calls in detail. For the most part, this results from the inherent fact that food safety is a multidisciplinary and highly complex field of research that is interwoven and overlapping with other research areas. As can be seen from the responses to question block 2, it is quite common for funding calls addressing individual aspects of food safety to be embedded in overarching themes such as agriculture, primary food production and/or processing, food security, diet & nutrition, or microbial & chemical hazards (Fig. 2), to name just a few examples. This circumstance renders it rather difficult for funding applicants to find the right call in the right place at the right time, but also for funding agencies among themselves to identify overlaps or duplications of funding activities. This situation is therefore certainly not conducive to fostering, for example, better coordination of funding programmes among different funding agencies at the national level or to promoting strategies for pooling resources and competencies among one another. However, it would be a big step forward, if food safety became recognized as an independent discipline/category in funding and tender opportunities, rather than being subsumed into other research areas. Similarly, it might be worth exploring the possibility of establishing specific national funding programmes dedicated to food safety, as a complement to the activities of the EFSA, and thus to further advance food safety research and development in the ERA.

III. Allocate food safety a priority state to accelerate the mobilization of research funding for bolstering scientific R&I

Evidence from the COVID-19 pandemic clearly demonstrated that during a global crisis situation, emergency research funding can be mobilized relatively quickly to explore solution strategies and advance basic research. The apparent increases in research funding were primarily attributable to intensified mobilization of research funders, who established rapid funding mechanisms but also prioritized pandemic-related research themes for funding to better target the evolving challenges [11]. The research system responded with vigor and flexibility during the pandemic, as did the research funding system and research infrastructures, which proved exceptionally capable of rapidly focusing on crisis-related problems and streamlining internal processes and expediting procedures. In addition, great emphasis was placed on prioritizing cooperative research projects and fostering rapid information exchange within the research communities and externally to provide a directional policy foundation. Even though most of the funding allocated to pandemic relief efforts was time-limited, it resulted in a strong push to take and advance science to the next level while demonstrating the effectiveness of this funding strategy. As can be seen from the answers to question block 3 (Fig. 3), a similar concerted crisis management dealing with food safety is not in place, but is highly desirable. From a global public health perspective, food is fundamental for all human existence and life without it is not feasible, making it one of the most important resources on earth. Especially healthy, nutritious and safe food should be the focus of our attention, and every effort should be made to ensure this condition in the long-term and on a global scale. However, numerous food scandals, such as feed and food emergencies in recent years or foodborne outbreaks, have highlighted shortcomings in addressing food safety threats and providing adequate resources to prevent or remedy irregularities that are harmful to humans. In addition, today's agrifood systems must evolve and adapt to major changes and challenges (e.g., climate change, resource depletion, dependence on global markets, environmental pollution, swelling populations, and food security) that affect our food systems and the human food chain, including food safety and quality. It is important and necessary to recognize that food safety is a shared and all-society responsibility, and it is now about time to depart from a mainly reactive approach to a proactive prevention mode by giving top priority to food safety programmes and food safety research activities. This also implies, that similar to the COVID-19 pandemic, concerted and coordinated efforts must be made by all countries, both within and beyond the EU, to increase financial resources and funding for food safety measures, ideally on a long-term basis complemented by follow-on financing of innovative and successful projects. This could effectively help to fill existing knowledge gaps, just as to prevent, detect and mitigate emerging and existing food-related risks and threats to human health. At the macro-level, newly acquired scientific evidence would help policymakers and state bodies to make informed decisions and/or enact new or amended policies and regulations, thus strengthening food safety systems at the legislative level, food control systems at the executive level, and helping to improve public health protection at the national and possibly global level.

IV. Raising private investment in food safety research

The topic of food safety is more important than ever today, especially in the current age of globalization and international interdependence with the mutual exchange of goods and foodstuffs. Food safety does not simply end at the borders of a single country, but affects us all at a transboundary level. In the face of the COVID-19 pandemic, attention turned to the vulnerabilities of our food systems and the need for sensible strategies to address them with great hope placed in scientific research. With a view to food safety, research funding is primarily provided by the EU or by EFSA, which is an independent agency funded by the EU to provide scientific and technical advice supporting evidence-based policymaking. As new problems rapidly emerge on the horizon, as is frequently the case in the food safety arena, supplementary funding sources not previously exploited are needed to provide a fast response. Given that food safety tends to be highly intertwined with the private sector (e.g., food industry, food business operators), it may be in an advantageous position to leverage extra funding from that side. In certain countries, as stated in the responses to question block 4 (Fig. 4), there is already the need for industry to co-fund research projects. The establishment of public-private partnerships (PPP) in particular has already proven to be effective [12], and might be an option to pursue more intensively. Evidence suggests that countries with more capacity to manage food safety risks have closer collaboration among the various public and private sector actors at play, along with being more proactive in developing and implementing partnerships. Dedicated PPP platforms in scientific research and food safety have already been established [12], linking various public and private sector stakeholders, which help to exchange information and views, just as to build mutual relationships and trust, thus encouraging in turn willingness to provide financial research support.

V. Improving transparency in the allocation of funding to distinct scientific disciplines

As indicated by the outcome of question block 5, there is a lack of transparency and evidence about how much funding is allocated or spent, for example, at national levels in individual EU MS/AC for the financing of research projects dedicated to food safety. Root causes in it are of various kinds. While the “major” funding bodies within individual EU MS are usually well known to researchers (e.g., ministries), there may exist complementary funding opportunities at national and regional levels provided by private organizations, foundations and charities, among others, which are often less well known. Frequently, no comprehensive overview or an all-embracing and up-to-date directory of all existing funders within a given country is maintained, or ideally accessible via a single gateway. The exchange of experiences with other projects (SCAR) and organizations (EFSA) unveiled similar difficulties in finding comprehensive information on national funders and identifying the right contacts at the various funding entities, given the extremely dispersed funding landscape in Europe. This fragmentation and information gap hinders a broad insight into the distribution of funding among scientific disciplines not assigned by governmental entities and impedes any thorough analysis. Secondarily, overlapping funding programmes from different funding agencies issued at the same times can diminish the effective use of already limited financial resources and thus unintentionally stimulate competition between researchers. It is well known that competition is an accelerator of scientific progress and innovation, and helps to prevent stagnation. However, competition that is too strong is seen as an impediment for transformative breakthroughs and the spirit of collaboration, diminishing maximum potential of exploitation.

VI. Prioritizing long-term developments and societal challenges for funding

The allocation of funding is usually directed towards previously identified target topics within individual funding entities. Research applications best satisfying the demands of funding providers are likely to get approval and financial support. This requires a certain amount of flexibility from researchers of always being at the leading edge of the relevant scientific topics available for funding. However, performing research and managing the underlying infrastructure planning processes are often very complex and time-consuming, especially with regard to research projects connected to the food safety field. Experience has demonstrated the demand for continuity and tenacity to develop target-oriented as well as activity-oriented approaches of tangible solutions. But still, the fast development of societal challenges and policy needs call for the rapid provision of scientific knowledge and approaches to solving existing problems. Political demands arising out of environmental, security and nutritional questions are changing today more quickly than scientists can respond to. By contrast, providing innovative solutions to the complex and pressing issues of our time makes it necessary to establish scientific knowledge resources and in developing creative, multidisciplinary and viable solutions, which require a certain run-up time. The current funding culture tends to be driven by a preference to fund quick, new, and sensational research results. Funding programmes for science should have some long-term stability and be developed with an eye to future developments and demands, rather than being subject to short-term political interests and dictates. Investment in food safety related research increases societies’ resilience towards crisis and prevents economical losses both within companies (e.g., food recalls) and society (e.g., foodborne diseases). In addition, consumer trust in the food value chain will increase.

VII. Need for provision of more long-term funding

Actual funding schemes are mostly designed for a relatively short period of time (in EU framework programmes like Horizon 2020 or Horizon Europe mostly 24–36 months). Given the complexity of societal challenges facing our societies today, a structured and more comprehensive approach is needed to address these wide-ranging challenges, calling for more long-term efforts. Scaling up the duration of research projects toward long-term funding of, say, 5–10 years, could foster an increase in effectiveness and impact, and thus generate both a greater profundity and a wider scope of effect. A longer funding horizon would in turn call for an augmentation of funds for covering extra expenditures. There are some research areas where long-term follow-up and data are needed such as for example exposure to toxins in food.

VIII. Transforming food safety research funding into a network of collaboration & dismantling bureaucratic hurdles

In conversation with scientists as the most frequent applicants for research support, the biggest obstacles in the acquisition of funding are widely seen in the availability of budgets that are too low for existing grants, high competitive pressure, and a long proposal development time with a simultaneous low success rate to be funded. Contrasting this with the insights gathered from the consultations with different funding bodies, most pressing challenges cited at their site were insufficient research funds (or shared resources with other agencies/ministries), along with a lack of workforce necessary for managing the funding calls (Fig. 6). As has already been discussed in a previous section, an increase in the amount of research & development spending might certainly be a desirable approach, acting as an important booster to advance food safety research and to drive innovation. A complementary and parallel course of action is needed in terms of reducing the administrative burden within national ministries or grant funding agencies associated with the reviewing and processing of submitted research applications. A limited number of funding calls commonly goes along with a high number of grant applications in food safety. The flood of incoming applications often does impose some challenges to the grant management offices, often limited in capacities and staff. A possible approach would be to transform grants management processes and systems into alliances established at national/regional levels, for instance through pooling of resources and/or by jointly developing more effective infrastructures for managing funding calls. In order to have these cooperative networks between different funding entities successfully translated into practice, internal procedures and working practices may require some adaptations and reorganization. Joining this process would further open up the opportunity to better exchange information on available funding opportunities in food safety and prioritized topics, gaining insights into funding workflows, funding cycles & grant periods, and funding instruments, just as to detect synergies between existing funds, funding resources and relevant research programmes at national/transnational/EU-level. Implementing an effective alignment of research funding in food safety requires as a precondition specialized knowledge of the subject, collaborative competencies and managerial capacities, along with an openness to transformational processes & capacity building, along with the willingness to explore unusual or previously unnoticed avenues on the part of the funding providers.

At this juncture, it is worth noting that the preceding recommendations by no means claim to be all-encompassing. They summarize the authors’ conclusions drawn from feedback gathered from a limited number of funding experts interviewed and workshop participants from a total of seven European countries (Belgium, Czech Republic, Finland, Germany, Italy, Portugal, and Romania). The views and recommendations expressed herein do not necessarily reflect those of the European Commission or other funding agencies not consulted.

## 5 Discussion and study conclusions

### 5.1 Policy implications

Nowadays, food systems are receiving more attention than ever before [13], not least due to the global COVID-19 pandemic, current crises and wars, and inherent challenges and vulnerabilities. In this context, policy debates and EU funded projects [14] widely employ terms such as food system transformation, healthy food and nutrition, food security, climate change, environmental protection, societal change, sustainability, resilience, food loss or waste and circular economy, among others. Evoking systemic change in food systems requires consideration and inclusion of multiple food system dimensions, including the provision of and access to sufficient food, the assurance of the safety of food, along with its availability and affordability. The general concept of food security is intimately linked to food safety albeit the latter is not always explicitly referenced. Somewhat out of the political spotlight, this has led to fewer and fewer research funding opportunities specifically tailored to food safety in recent years. For example, under Horizon 2020, out of a total of 586 calls for research proposals in the area of food systems, with a total allocated budget of €3062 million, only 22 calls (i.e. 4 %) were funded on the topic of food safety. In contrast, the research categories of primary production and food processing ranked significantly higher, with 279 (48 %) and 150 (25 %) research calls, respectively [15]. Even Horizon Europe provides an indicative total budget of €8952 million for the programme cluster 6 geared towards “Food, Bioeconomy, Natural Resources, Agriculture & Environment” and is thus three times as high as in Horizon 2020 [16], a closer look at the destinations and impact areas of this cluster reveals that food safety is not explicitly listed or is only touched upon tangentially in the tenders [17]. However, food safety research plays a vital role in driving innovation by detecting potential hazards and devising strategies to prevent foodborne illnesses and safety concerns. Adequate funding for food safety research can enhance our knowledge of the underlying causes of food contamination and pave the way for novel technologies, practices, and regulations to mitigate risks and augment food safety. These efforts can lead to the development of innovative products, services, and practices that are safer, more efficient, and eco-friendly. In addition, food safety research can instill consumer confidence in the safety and quality of the food supply, which is essential for public health and economic growth. Food safety thus holds political significance as it directly affects the health and well-being of the global population. Therefore, policy makers need to take necessary measures and allocate adequate resources to promote food safety, including research funding, to mitigate the risks associated with unsafe food and protect public health. The study at hand shows that the research funding landscape in food safety is facing diverse and far-reaching problems. Not only a need for adjustment at the monetary level was detected, but also a need for reforming organizational and infrastructural processes within national funding agencies was uncovered. Furthermore, there is a demand to expand the scope of current collaboration efforts and actively seek out and advance opportunities for networking at an international level. The establishment of worldwide networks among research funding actors in the area of food safety is a critical prerequisite for ensuring sustainable food security and resilience of food supply chains both within and outside Europe. In order to trigger large-scale systemic changes in funding structures in the long term, political discussions and steering measures in cooperation with research funders are needed to initiate a transformation process of the currently rather rigid European research support systems.

### 5.2 Ongoing transformational changes in european food safety funding structures

Within the current 9th Research Framework Programme Horizon Europe (2021–2027), the EU is moving towards the implementation of European Partnerships as long-term instruments for concerted multidisciplinary actions amongst private and/or public partners in R&I as a strategy to more effectively address the pressing challenges of our time [18]. The European Partnerships are thus replacing *trans*-national funding schemes like the ERA-NETs. The European Partnerships are not only an important pillar of Horizon Europe but are also expected to contribute significantly to the implementation of the EU's political priorities and missions. Particular advantages of the European Partnerships are their capacity to better coordinate, align and leverage European and national R&I efforts, which is being taken forward with the setting of thematic research priorities in the framework of newly established multi-stakeholder and long-term research agendas, and enhanced cross-Partnership dialogue and cooperation. The operational mandates of the 49 envisaged European Partnerships [19] are directed towards the following Sustainable Development Goals (SDG) adopted by the United Nations MS in 2015 as part of the 2030 Agenda for Sustainable Development: 1) *SDG3*: Good health and well-being; 2) *SDG8*: Decent work and economic growth; 3) *SDG9*: Industry, innovation and infrastructure; 4) *SDG11*: Sustainable cities and communities; 5) *SDG12*: Responsible consumption and production; 6) *SDG13*: Climate action. Particularly in view of the *SDGs No*. *3, 8, 9, 11* and *12*, attention is also being drawn to food safety, whose interrelationships and implications for individual SDGs have been described in more detail elsewhere [20]. Concerning the subject of food safety, preparations of the EU are now underway for establishing a new European Partnership on ‘Sustainable Food Systems for people, planet and climate' within cluster 6 targeting ‘food, bioeconomy, natural resources, agriculture and environment'. The stated mission of this Partnership is to speed up Europe's food systems transformation towards more *‘healthy diets and food that is safe and sustainably produced in resilient EU and global food systems’* [13]. The SFS Partnership is planned to be launched in 2023 and will merge existing R&I funding schemes such as ERA-NET Cofunds under Horizon 2020 into a single funding instrument [19]. On an indicative basis, the SFS Partnership will operate for seven to ten years and will be co-funded by the EU and its MS/AC with a total planned budget of approximately €175 million, topped-up by financial or in-kind commitment from private (e.g., private for-profit companies) and other partners [7].

Applying a systemic approach, the SFS Partnership will address the following key issues related to the food systems environment: 1) Change the way we eat; 2) Change the way we process and supply food; 3) Change the way we connect with food systems; and 4) Change the way we govern food systems [8]. The preparation for the establishment of the SFS Partnership and its SRIA under the leadership of SCAR Food Systems is still ongoing [8], supported by the Horizon 2020 project *FOODPathS* (https://www.foodpaths.eu), among others. The expectations for the SFS Partnership are quite high, but at the same time, it will open up new opportunities to address and solve contemporary challenges in a way that individual EU-funded R&I projects or initiatives with limited scope, budget, and human resources, have not been able to do so far on a stand-alone basis and to the extent needed. Major benefits of the new SFS Partnership are certainly that it does not operate in isolation, nor is it limited to a specific number of participants, but is open to a wide range of actors and stakeholders, throughout its lifetime [19], while also building on existing knowledge and expertise to derive maximum synergy gains. This will not only foster innovation and advancements in research and development, but also increase the necessary clout and power needed to address existing and forthcoming societal challenges in a more strategic and eye-to-eye manner on an international scale. Due to its targeted mission and impact-oriented approach, the SFS Partnership will also serve to mobilize a critical mass in human and financial resources (public and private investment) in the longer term and to further develop a knowledge base and capacities at the national and *trans*-national level by building networks and collaborations with, for example, European research infrastructures, Innovation Hubs, or Cluster Initiatives [21]. This may well be a door-opener to accessing alternative instruments, (national) funding and/or investments needed to further enhance the potential of the SFS Partnership and increase research activities, performance and innovation capacities and thereby accelerating the transformation of the European food systems toward greater safety and sustainability.

## 6 Limitations of the study

The first step in identifying solutions to existing problems associated with research funding involves an in-depth review of the current situation in individual EU countries. On a practical level, this has proven challenging in some places, as funding donors are not always cooperative in their willingness to share internal information with the outside world in a voluntary manner. Given time and resource constraints for the study at hand, which was conducted as part of a Social Lab piloting exercise, the research design was limited to a small number of expert interviews, with particular emphasis on gathering in-depth information from at least two different countries per FS4EU Hub. We are well aware that this small sample size is not representative for all EU MS/AC and does not necessarily reflect the full range of issues related to research funding in all European countries. This certainly requires a wider investigation, in an optimal setting with each national focal point as a central point of contact, in order to more efficiently channel and force the exchange of knowledge and information collection. In this context, it should be noted that the challenges faced by different funding agencies within a single country can vary widely and require differentiated stocktaking. Overall, the present study has uncovered a number of significant existing problems in the context of research funding and identified possible ways to overcome them. However, it can only appeal to a funding organization to address the issues raised by experts, without actively driving this process in any way. Rather, the recommendations presented herein can only provide a gentle ‘theoretical' nudge to achieve the overall goals. Putting the advice into practice is entirely at the discretion of individual funding agencies themselves, and it is up to them to adopt any changes.

## 7 Summary & outlook

The results of this survey should help to improve understanding of the funding mechanisms and frameworks applied in the different EU countries, as well as any differences in procedures or strategies followed. Reflecting on the recommendations outlined in the previous section with regard to the identified shortcomings in food safety research funding, European Partnerships integrate quite fitting solutions for some of the aforementioned problems and mark an important milestone. In general, European Partnerships offer the opportunity to effectively advance topic-specific scientific activities of particular brisance for the individual and society and in the sense of a pro-active prevention principle in coordinated actions deliberately and over a prolonged period of time. The establishment of a specific European Partnership with a particular focus on food systems that is inextricably linked to food safety also underscores the importance of this specific scientific field and highlights an existing or urgent need for action and research, which simultaneously implies the existence of some shortcomings in this regard.

The targeted provision of funding by the EU and other investors, as a means of boosting scientific research and innovation, is also an important and promising prerequisite for addressing the major societal challenges of our time across scientific disciplines. The extent to which the aspired interdisciplinary multi-actor and collaborative approach of the European Partnerships will provide the impetus for greater allocation of resources of a financial, human, and technical nature remains to be awaited for the time being, until the SFS Partnership gets operational. Overall, European Partnerships will open up many new opportunities to overcome identified barriers and fragmentation encountered in the area of research funding, including those related to food safety.

Looking ahead, the knowledge gained from our pilot study offers for the first time more in-depth insights into what research funding experts themselves consider as major problems and challenges in the European research funding environment, and which remedial strategies should be considered sensible or aimed at. We hope that our results will contribute to improve the European funding situation by encouraging better dialogue and communication between different funding organizations on the national and/or *trans*-national level. An immediate step taken by FS4EU to drive positive change is the establishment of a novel EU Food Safety Forum (to be launched in November 2023), as a long-lasting and sustainable multi-stakeholder platform. It is intended as a meeting and information place for various food safety actors, inter alia funding authorities, to exchange, discuss, and better synchronize activities and research strategies through a participatory process, and as a science-policy-society interface. A regular exchange of experiences and information is an important first step to make internal processes more transparent and to overcome existing fragmentation by bundling capacities and creating collaborative networks among research funders in the longer term. Better coordination and pooling of resources, as well as greater use of synergies, will help strengthen the ERA and its ambition to better align and streamline public research funding through the establishment of joint programmes and global partnerships [22].

## Funding statement

This work was funded by the European Union's Horizon 2020 Research and Innovation programme (H2020-EU.3.2.2.2. – Healthy and safe foods and diets for all) [grant number 101000613] as part of the FoodSafety4EU project (Call: H2020-FNR-2020-1).

## Data availability statement

All data generated or analysed during this study are included in the published article and were not deposited into a publicly available repository.

## CRediT authorship contribution statement

**Tamara Stelzl:** Writing - review & editing, Writing - original draft, Visualization, Resources, Project administration, Methodology, Investigation, Conceptualization. **Nastasia Belc:** Writing - review & editing, Resources, Project administration, Methodology, Investigation, Conceptualization. **Nunzia Cito:** Writing - review & editing, Resources, Project administration, Methodology, Investigation, Conceptualization. **Veronica M.T. Lattanzio:** Writing - review & editing, Resources, Project administration, Methodology, Investigation, Conceptualization. **Celine Meerpoel:** Writing - review & editing, Resources, Project administration, Methodology, Investigation, Conceptualization. **Sarah De Saeger:** Writing - review & editing, Resources, Project administration, Methodology, Investigation, Conceptualization. **Hanna-Leena Alakomi:** Writing - review & editing, Resources, Project administration, Methodology, Investigation, Conceptualization. **Monika Tomaniova:** Writing - review & editing, Resources, Project administration, Methodology, Investigation, Conceptualization. **Jana Hajslova:** Writing - review & editing, Resources, Project administration, Methodology, Investigation, Conceptualization. **Sophie Scheibenzuber:** Writing - review & editing, Writing - original draft, Visualization. **Michael Rychlik:** Writing - review & editing, Writing - original draft, Visualization, Resources, Project administration, Methodology, Investigation, Conceptualization.

## Declaration of competing interest

The authors declare that they have no known competing financial interests or personal relationships that could have appeared to influence the work reported in this paper.
