# Supplementary material for: European food safety research: An explorative study with funding experts’ consultation
Source: Heliyon. 2023 Nov 28;9(12):e22979. doi: 10.1016/j.heliyon.2023.e22979 (PMC10731067; doi:10.1016/j.heliyon.2023.e22979)
Supplement: Multimedia component 1 [file mmc1.pdf]

Supplementary Material

European food safety research: An explorative study with funder consultation

Tamara Stelzl[a], Nastasia Belc[b], Nunzia Cito[c], Veronica M. T. Lattanzio[c], Celine Meerpoel[d], Sarah De Saeger[d], Hanna-Leena Alakomi[e], Monika Tomaniova[f], Jana Hajslova[f], Sophie Scheibenzuber[a, *], Michael Rychlik[a]

[a]Technical University of Munich, TUM School of Life Sciences, Department of Life Science Engineering, Chair of Analytical Food Chemistry, Maxiumus-von-Imhof-Forum 2, 85354 Freising, Germany; tamara.stelzl@tum.de; sophie.scheibenzuber@tum.de; michael.rychlik@tum.de

[b]National R&D Institute for Food Bioresources, IBA Bucharest, Banesa Ancuta 5, 021102 Bucharest, Romania;
nastasia.belc@bioresurse.ro

[c]National Research Council of Italy (CNR), Institute of Sciences of Food Production (ISPA), Via Amendola, 122/O, 70126 Bari, Italy;
nunzia.cito@ispa.cnr.it; veronica.lattanzio@ispa.cnr.it

[d]Ghent University, Department of Bioanalysis, Centre of Excellence in Mycotoxicology and Public Health, Ottergemsesteenweg 460, 9000 Ghent, Belgium;
celine.meerpoel@Ugent.be; Sarah.DeSaeger@Ugent.be

[e]VTT Technical Research Centre of Finland Ltd., P.O. Box 1000, FIN-02044 VTT, Finland; hanna-leena.alakomi@vtt.fi

[f]University of Chemistry and Technology Prague, Faculty of Food and Biochemical Technology, Department of Food Analysis and Nutrition, Technicka 3, Prague 6, 166 28, Czech Republic;
Monika.Tomaniova@vscht.cz; Jana.Hajslova@vscht.cz

[*]Corresponding author

E-mail address: sophie.scheibenzuber@tum.de (S. Scheibenzuber).

Questionnaire

*****_Required_

0.1 Which country are you from? *

Open response.

____

0.2 Which is your affiliation? *

Mark only one oval.

- ☐ Public/Government Funding Agency / body
- ☐ Private company
- ☐ Foundation
- ☐ Other: _______

1. **Which organisation(s) is/are primarily in charge to allocate budget in your country (at national level) for Food Safety related research?** *

   Open response.

   ____

2. **Who is involved in the decision-making process of allocating funds to your national programme? (e.g., technical tables, advisory boards, independent experts)** *

   Tick all that apply.

   - ☐ Ministry
   - ☐ Academia
   - ☐ Research
   - ☐ Private sector - Foundation
   - ☐ Private sector - Industry
   - ☐ Private sector – (Agri-)Food Associations
   - ☐ Other: _______

3. A) Indicate up to three of the most influential entities/activities for identifying and prioritizing research topics for being funded in your country! *

Tick maximum 3 ovals.

- ☐ SRIA – Strategic Research and Innovation Agenda
- ☐ RIS3 (National Research and Innovation Strategy for Smart Specialisation)
- ☐ European Agendas
- ☐ EFSA opinions, EFSA Forum
- ☐ Expert discussion rounds (e.g., ad-hoc advisory boards)
- ☐ Thematic Research Programme
- ☐ Public-private associations (national & regional clusters)
- ☐ Working groups
- ☐ Public consultations
- ☐ Committees
- ☐ Internal discussion (governmental level)
- ☐ Other: _______

B) How do you identify & prioritize research topics for being funded? *

Tick all that apply.

- ☐ Top-down approach
- ☐ Bottom-up approach
- ☐ Other (i.a. competitive calls, agri-food clusters): _______

4. Which are the main fields in which the Food Safety is embedded in your country?

Tick all that apply.

- ☐ Agriculture (primary production)
- ☐ Farming practices
- ☐ Fishery
- ☐ Breeding/livestock
- ☐ Food processing
- ☐ Diet, nutrition
- ☐ Consumer behaviour
- ☐ Communication & social initiatives
- ☐ Sustainability & circular economy
- ☐ Digitalization
- ☐ Aquaculture
- ☐ Food security
- ☐ Chemical & microbial hazards
- ☐ Foodborne illnesses
- ☐ Emerging contaminants
- ☐ Analytical tools for monitoring purposes
- ☐ Genetically modified organisms (GMO)
- ☐ Zoonotics in primary production

5. Please select the scale of funding you usually provide for Food Safety related research activities/calls.*

Tick all that apply.

- ☐ National
- ☐ Regional (at EU level: Central, Eastern, Western, Southern Europe)
- ☐ International (i.e. export)
- ☐ Local (including regional level in a country)

6. Which type of actions do you fund primarily in the Food Safety area? *

Tick all that apply.

- ☐ Research and innovation actions (RIA), ERA-NETs
- ☐ Innovation actions (IA)
- ☐ Coordination and support actions (CSA, COST actions) at international level
- ☐ Collaborative action/networking at national level
- ☐ Basic research
- ☐ Applied research
- ☐ Experimental research
- ☐ Industrial research
- ☐ Collaborative research
- ☐ Contractual research
- ☐ Technology transfer
- ☐ Communication
- ☐ Education and training
- ☐ Policy
- ☐ Other: _______

7. How do you react to emerging and crisis situations?
(e.g., brief description of any changes in decision-making processes)

Open response.

____

8. If there is any emerging and crisis-related action, is there allocated extra funding for rapid actions and needs?

Mark only one oval.

- ☐ Yes
- ☐ No

9. On average, how many projects in the area of Food Safety do you fund per year (also including areas in which Food Safety is embedded)?

Mark only one oval.

- ☐ <10
- ☐ 10-50
- ☐ >100
- ☐ not applicable (in case of unavailable information)

10. On average, how much do you spend annually for the financing of research projects in the Food Safety field (in million Euro)?

Mark only one oval.

- ☐ 0.1
- ☐ 0.3
- ☐ 0.5
- ☐ 1
- ☐ 1.5
- ☐ 2
- ☐ 2.5
- ☐ 3-9
- ☐ 10-15
- ☐ >15

11. Is co-funding needed for project proposals? *

Mark only one oval.

- ☐ Yes
- ☐ No
- ☐ For some of them

12. If co-funding is needed, who usually co-funds proposals? (e.g., industry, other types of organisations)

Open response.

____

13. Do you collaborate with other funders in your country or do you harmonize themes and/or the publication timing of your funding calls against each other (consolidated approach)? *

Open response.

____

14. Do you also collaborate with funders from other countries and if yes, with which?

Open response.

____

15. What do you see as the biggest challenges in your funding agency with regard to the implementation of research funding matters? *

Tick all that apply.

- ☐ Insufficient research funds (or shared resources with other agencies/ministries)
- ☐ Insufficient workforce for management of the calls
- ☐ Short deadlines for calls
- ☐ Grant proposal do not comply with the formal requirements of the funder
- ☐ Ad hoc requests for the implementation of funding calls from government agencies
- ☐ High bureaucratic burden
- ☐ Lengthy run-up times before a funding call can be launched (e.g., procedural obstacles)
- ☐ Food and related topics not being a priority for funding
- ☐ Proposal lacking fit for purpose (e.g., in terms of scientific quality, novelty)
- ☐ Too many grant applications received (hard decision-making)
- ☐ Thematic divergence of a submitted grant proposal from a call text
- ☐ Other: _______
